# Incidence of diabetic retinopathy in anti-tnf treated rheumatic disease patients with type 2 diabetes

**DOI:** 10.1007/s00417-024-06529-3

**Published:** 2024-06-06

**Authors:** İffet Merve Uçar Baytaroğlu, Ata Baytaroğlu, Merve Uçar Toros, Hatice Daldal

**Affiliations:** 1 Rheumatology Department, Uşak Training and Research Hospital, Uşak, Turkey; 2 Ophthalmology Department, Uşak Training and Research Hospital, Uşak, Turkey; 3 Endocrinology Department, Uşak Training and Research Hospital, Uşak, Turkey

**Keywords:** Anti-TNF, Rheumatic disease, Diabetes complications, Retinopathy

## Abstract

**Objective:**

This study aimed to evaluate the impact of anti-TNF (biological) therapies on the incidence and progression of diabetic retinopathy.

**Materials and methods:**

A cross-sectional analysis of 50 diabetic patients with rheumatic diseases (group 1) was performed. An age-, sex-, and HbA1c-matched control group (group 2) was formed from a pool of diabetic patients who underwent regular eye examinations. The presence or absence of diabetic retinopathy was also assessed. Comorbidities such as hypertension, coronary artery disease, and hyperlipidemia were also evaluated as possible confounding factors.

**Results:**

Hundred eyes of 50 patients were evaluated in each group. Only three patients in group 1 had non-proliferative retinopathy. The median duration of rheumatic disease was 9 years, whereas that of diabetes was 11 years. The mean duration of anti-TNF therapy was 4 years. In the control group of diabetes-only patients, 13 patients developed some form of newly diagnosed diabetic retinopathy during the last five years. The calculated retinopathy occurrence between the groups was statistically significant (*p* < 0.05). In this study, the incidence rate ratio for patients receiving anti-TNF treatment was calculated as 0.4 in the study.

**Conclusion:**

TNF inhibitors, with their anti-inflammatory effects, positively impact diabetic complications by reducing the incidence of retinopathy. To our knowledge, this is the first study to evaluate retinopathy development after anti-TNF therapy.

## Key messages

***What is Known:***

• Inhibition of TNF? is known to be beneficial in reducing the risk of diabetes itself in rheumatic disease patients.
• Disorders related to TNF? and its pathways are critical contributors to microvascular complications, such as prolif?erative retinopathy.

***What is New:***

• Our preliminary data shows that TNF inhibitors positively impact retinal microvasculature, lowering diabetic reti?nopathy incidence.
• Glycemic control is potentially the most critical parameter of the development of diabetic complications in rheumatic disease patients, even under effective anti-inflammatory treatment.

## Introduction

The role of anti-inflammatory drugs in treating diabetes and its complications has been a focal point of diabetes research since the 1870s [1]. The novel findings of animal studies demonstrating that TNF-α has a direct role in the pathogenesis of diabetes and insulin resistance have opened the door for new studies in this field [2]. However, dietary habits and lifestyle changes throughout the century have resulted in a quarter of the global population developing diabetes [3, 4]. Since the annual incidence of retinopathy development and progression in diabetic patients is–2–12%, diabetic retinopathy (DR) has become one of the costliest visual impairments worldwide [5]. Although TNFα is still a critical factor in the pathogenesis of diabetic retinopathy, among other inflammatory mediators, controversial data restrict its widespread use [6, 7]. Impaired TNFα production is an essential factor leading to microvascular complications, especially proliferative diabetic retinopathy [8]. Currently, the main treatment options are intravitreal use of anti-vascular endothelial growth factor (VEGF) and pigment epithelium-derived factor (PEDF) agents [3, 5]. 

Infliximab and adalimumab, the first FDA-approved anti-TNF-α agents, have been used in various rheumatic and dermatologic disorders, such as rheumatoid arthritis (RA), psoriasis, psoriatic arthritis (PsA), and ankylosing spondylitis (AS), for over two decades with proven efficacy and safety [9]. TNF-α antagonism has also been shown to reduce the incidence of DM in these patients by nearly halving the risk of diabetes [10]. To our knowledge, the incidence and severity of diabetic retinopathy in patients receiving anti-TNF treatment have not been evaluated.

In this study, we examined the effect on the development of diabetic retinopathy by reviewing the course of cases treated with biological agents (adalimumab, infliximab, etanercept, golimumab, and certolizumab), which are the first choice in rheumatology practice in patients unresponsive to conventional therapies.

## Materials and methods

We screened 2558 diabetic patients (1617 male and 941 female) who were followed up with OCT in our hospital. Patients with no retinopathy at baseline evaluation were selected, including OCT and a thorough clinical examination. Similarly, patients with rheumatologic diseases with detailed baseline eye examinations were scanned. Patients with overlapping diagnoses of diabetes and rheumatic diseases were analyzed, and the subjects treated with TNFi were recruited. After applying the exclusion and regular follow-up criteria, the remaining 121 diabetic subjects without known rheumatic disease were randomized according to age, gender, and HbA1c criteria to be matched with the TNFi-treated cases.

We managed to analyze the data from 100 eyes of 50 patients with type 2 diabetes who were taking biologics for various rheumatic diseases. As meta-analyses have shown, the most determinant factors associated with diabetic retinopathy are HbA1c levels, nephropathy, lipid profiles, and cardiovascular status [11, 12]. Therefore, to compare and evaluate retinopathy development, we randomly chose age-(± 3 years), sex-, and mean HbA1c-matched diabetic patients without any rheumatic disease and previous evidence of retinopathy who were followed up with spectral-domain optical coherence tomography (OCT) in our clinic. Randomization could not be performed in cases where only one case was eligible for matching. Since rheumatology patients come to follow-up more regularly, we also matched the average number of endocrine/internal medicine visits per year to avoid causing Berkson bias by skipping non-complicated diabetic patients with fewer hospital admissions. We did not include patients with a body mass index (BMI) > 30 and glomerular filtration rate < 30 mL/min/1.73 m^2^. The remaining exclusion criteria were the presence or sequelae of uveitis, history of intraocular surgery or ocular trauma, chronic steroid use (systemic and/or ocular), glaucoma, age-related macular degeneration, retinal vascular occlusion, and dense cataracts obscuring the OCT image quality. Patients with a history of long-term hydroxychloroquine use (≥ 5 years) were also excluded. None of the patients had any other accompanying systemic or ocular diseases, except for the comorbidities examined.

Demographic features, including age, sex, rheumatic disease diagnosis and duration, diabetes course, anti-TNF treatment and duration, and any accompanying diseases were noted. In addition to routine ophthalmic evaluation, patients were examined, and average foveal and macular thickness measurements were obtained with OCT (HS100 RX Capture v.4.5, Canon Europe, Netherlands) at least once a year. All patients were followed up and classified according to the International Council of Ophthalmology Diabetic Retinopathy guidelines [13]. Fundus fluorescein angiography (FFA) was also conducted on a clinical basis. All subjects underwent comprehensive dilated initial eye examinations following the diagnosis of type 2 diabetes. All HbA1c values included in the study were measured by ion-exchange high-performance liquid chromatography (HPLC) using a Bio-Rad Variant Turbo instrument (Siemens).

The sample size was calculated using G*Power Version 3.1.6. In Turkey, Taş et al. [14] conducted a multicenter study on the epidemiology of DR in 2362 patients and found a DR prevalence of 30.5%. Considering the difference as a threefold drop in the expected incidence of DR in Group 1, the sample size was calculated as approximately 49 in the groups and 98 patients in total for an alpha significance level of 0.05 for 0.8 power.

PASW for Windows (version 15.0, SPSS Inc., Chicago, IL, USA) was used for the statistical analyses. Descriptive statistics were presented as numbers and percentages for categorical variables and medians and interquartile ranges for numerical variables. The proportions in the groups were compared using the chi-squared test. Because continuous variables did not follow a normal distribution, two independent group comparisons were made using the Mann–Whitney U test. Risk factors were analyzed using a logistic regression analysis. The statistical significance level was set at *P* < 0.05.

## Results

There was no significant difference between the two groups regarding age, gender, and duration of diabetes. There was also no significant difference between the groups regarding glycemic control, renal function, or other comorbidities. The demographic features of both groups are summarized in Table 1.

**Table 1:** Demographics and characteristics of the studied groups

	Group-1	Group-2	*p*-value
Age*	59.5 (55–63.25)	56 (48–66)	0.38
Gender (M: F)*	22/28	22/28	
Creatinine (mg/dL)	0.77 (0.68 − 0.9)	0.805 (0.72 − 1)	0.15
Rheumatic Disease Distribution			
Rheumatoid Arthritis Ankylosing Spondylitis Psoriatic Arthritis	24 (48%) 20 (40%) 6 (12%)		
Anti-TNF agents			
Adalimumab Infliximab Etanercept Golimumab Certolizumab	14 (28%) 12 (24%) 14 (28%) 5 (10%) 5 (10%)		
Rheumatic Disease Duration (years)	9 (5.75 − 12)	N/A	
Anti-TNF Duration (years)	3.5 (2–5)	N/A	
Diabetes Duration (years)	10 (5–19.25)	11 (6–15)	0.78
HbA1c value (%)*	7.5 (7.1–8.1)	7.35 (6.8–8.15)	0.61
Hypertension (%)	13	9	0.33
Coronary Artery Disease (%)	22	20	0.80
Hyperlipidemia (%)	28	34	0.51

*: Matched parameters

**: A *p* value lower than 0.05 was considered significant

In the rheumatic disease group (group-1) twenty-four (48%) patients were diagnosed with rheumatoid arthritis, 20 (40%) with ankylosing spondylitis, and 6 (12%) with psoriatic arthritis. Fourteen patients were treated with etanercept or adalimumab. Twelve patients received infliximab, and five patients each were taking golimumab and certolizumab, and the median rheumatic disease duration was 9 (6–12) years. The median duration of TNFi use was 3.5 (2–5) years.

The retinal evaluation revealed median foveolar thickness and macular thickness as 255 μm (245–274) and 301 μm (286–315) in group 1 compared to 264 μm (246–278) and 295 μm (286–310) in group 2 (*p* = 0.28/*p* = 0.71). In approximately 5 years of retinal follow-up, non-proliferative DRP developed in only 5 of 100 eyes (3 patients), and macular edema was found in only one eye treated with focal laser photocoagulation (Table 2). Retrospectively, 43% (21) of the patients had FFA in group 1, but none showed any evidence of proliferative retinopathy. However, in Group 2, 24 eyes of 13 patients developed some form of diabetic retinopathy during the last five years. Nine patients had background diabetic retinopathy changes such as microaneurysm, hard exudate, or dot-blot hemorrhages, and four patients were diagnosed with diabetic macular edema and needed intravitreal treatments.

**Table 2:** Retinal features of the studied groups

	Group-1	Group-2	*p* value
Macular thickness (µm)	301 (286–315)	295 (286–310)	0.71
Foveal thickness (µm)	255 (245–274)	264 (246–278)	0.28
Presence of diabetic retinopathy	3/50 (6%)	12/50 (26%)	0.006*
Non-proliferative changes Proliferative changes Macular edema	5/100 (5%) None 1/100 (1%)	17/100 (17%) None 7/100 (7%)	0.007* - 0.03*

*: A *p*-value lower than 0.05 was considered significant

Although it is noteworthy that all patients with retinopathy in both groups had a diabetes duration of ≥ 5 years, it was calculated that the duration of diabetes did not significantly affect the development of retinopathy in our study (*p* = 0.09). However, diabetes duration had a weak but statistically significant positive correlation with macular thickness in both eyes (*p* = 0.04/*p* = 0.013/r:0.25). DR development was significantly higher in patients with HbA1c > 7.45 when all cases were considered (*p* = 0.006). In the univariate analysis of the risk factors for DR, an increase in HbA1c and creatinine levels were found to be a risk factor, whereas TNF inhibition was found to be a protective factor (*p* < 0.001 *p* = 0.020 *p* = 0.012). In the multivariate logistic regression analysis (Table 3), only increased HbA1c level was seen as a risk factor, but the protective effect of TNF inhibition was sustained (*p* < 0.001, *p* = 0.01). A chi-square test was conducted to evaluate the occurrence of retinopathy, which yielded statistically significant results (*p* = 0.006). The incidence rate ratio in our study was calculated as 0.4 in our study.

## Discussion

The lower risk rate calculated in the anti-TNF treatment group supports the basis of intravitreal drug studies in this field [9]. In a series of longstanding DM cases over 20 years, high TNFα receptor levels were particularly associated with developing proliferative changes. As the disease progressed, TNFα production doubled and was correlated with the severity of retinopathy [8, 15]. Our study with a limited sample size investigated the effects of TNF inhibitors for an average of 4 years. Although the groups did not differ in retinal thickness measurements, vascular changes, or related findings, it revealed an increased incidence of diabetic retinopathy.

TNF inhibitors are the mainstay treatment options for autoimmune and autoinflammatory diseases, and autoimmunity is predominantly a feature of type 1 DM. However, inflammation and insulin resistance are observed at different rates in both types of diabetes. The development of long-term organ-damaging complications in type 2 DM is of genetic origin and exhibits wide heterogeneity [16, 17]. 

The role of TNF in diabetes complications has already been elucidated; for example, increased TNFα levels have been observed in tear samples of diabetic patients, which has also been shown to correlate with retinopathy and nephropathy severity [18]. Another suggested mechanism of action of TNF is that increased TNFα during the inflammatory process in chronic diabetes may cause antibody-mediated retinal pericyte damage by upregulating CD38 [19]. 

Supporting the role of TNF-α in the pathogenesis of diabetes, cohorts receiving anti-TNF-α therapy, particularly infliximab, have shown significantly lower mean blood glucose levels and reduced insulin resistance. A retrospective study with limited subjects showed that infliximab had a 1% effect on HbA1c levels in the long-term. Nevertheless, the low mean HbA1c level in this study is an important limiting factor [20]. In a recent study, the blood glucose-lowering effect of adalimumab was found to be more pronounced in the psoriatic group than in the rheumatic disease group [21–23]. Several mechanisms have been reviewed regarding the glucose-lowering effects of biologics, including reduced TNFα-induced insulin resistance, reduced soluble ICAM1 levels, and increased high-molecular-weight adiponectin [24]. Unfortunately, due to the limited number of patients treated with each anti-TNF agent, we could not demonstrate their effects on glucose levels and the incidence of retinopathy separately. We aimed to homogenize our study group in terms of HbA1c levels rather than compare their current blood glucose levels or diabetes treatments, because we wanted to evaluate the long-term effects of TNF inhibitors. In our study, an HbA1c level of 7.45, despite being low, was found to be an independent risk factor for the development of DR. However, a slightly higher proportion of patients in group 2 using insulin may contribute to increased retinopathy, necessitating tighter glucose control to achieve lower HbA1c percentages. Furthermore, some antihyperglycemic drugs such as pioglitazone have been linked to an increase in diabetic macular edema [25]. However, we assumed that drug-related cases would not be overlooked because there was no significant difference in macular thickness between the groups in our study, and retinal evaluation was already performed with detailed fundoscopic imaging.

Although only 3 patients developed diabetic retinopathy under anti-TNF treatment, we believe that it should not be overlooked that anti-TNF agents activate different autoimmune disease pathways in addition to their presumed protective effects. This TNF inhibitor-induced autoimmune disease spectrum covers a wide range, from mild serologic changes to a full-blown systemic autoimmune disease, causing severe end-organ damage [26]. Infliximab has been shown to cause ANA seropositivity in more than half of cases after the initiation of treatment [27]. Systemic and retinal vasculitis induced by anti-TNF treatment, with or without accompanying retinal vein occlusion, has been reported in approximately 4% in some series [28]. Uveitis and optic neuritis have also been reported as the potential side effects of these treatments. Furthermore, the incidence of DM under anti-TNF therapy from large-scale US data was approximately 6% per 1000 person-years. This effect was more prominent in the infliximab and adalimumab groups than in the abatacept group. They also stated that baseline obesity might be a critical factor contributing to the development of DM with biologics [29]. We did not consider BMI as a confounding factor, as we excluded patients with BMI > 30; however, borderline overweight subjects might have created a bias in favor of a higher retinopathy incidence in group 2. However, a 20-year survey revealed that being slightly overweight did not increase the risk of DM complications, particularly in men [30]. Another potential bias in our study is the occasional and previous use of DMARDs, NSAIDs, and steroids used for acute exacerbations. However, since chronicity is an essential factor in the effect of autoimmunity on the course and complications of diabetes and animal studies have shown that NSAIDs only show anti-TNF-like TNF blockade when used in high doses, we excluded acute exacerbation treatments [31, 32]. But perhaps even more importantly, since anti-TNF therapies were not first-line drugs in any of the diagnostic groups included in the study, it must be accepted that first-line therapies also have a certain level of anti-inflammatory effect. However, when we look at the studies in the literature examining the impact of DMARD use on the development of diabetes in rheumatic disease patients, it is noteworthy that this effect is only evident within the first 2 years of treatment, and the risk reduction is mainly in patients aged > 60 years [33]. This again reiterates that chronicity is a vital determinant in the process.

Apart from their systemic use, etanercept, adalimumab, and infliximab have been trialed as intravitreal therapeutic agents for various inflammatory conditions. Etanercept, which has a shorter half-life than the other drugs, works as a trap receptor, binds to TNF1-2 receptors, and inhibits TNF-mediated pathways. Although its intraocular anti-inflammatory effect has been evaluated and shown to reduce PVR in rabbit eyes, no successful outcomes have been observed in human studies of diabetic retinopathy [9, 34, 35]. Adalimumab, a human monoclonal antibody in IgG1 form, has been FDA-approved since 2002. Arevalo et al. achieved three ETDRS lines of visual gain in a series of seven patients in whom the intravitreal use of adalimumab combined with bevacizumab (anti-VEGF) was tested [6]. Infliximab, another monoclonal IgG1 antibody with a chimeric structure, has been tested in various diseases such as wet age-related macular degeneration, posterior uveitis, and diabetic macular edema. However, except for a 1-month transient effect in chronic uveitis, especially in Behçet’s disease, and up to a 45% reduction in macular edema in a limited number of cases unresponsive to focal therapy, it failed to provide an established impact in long-term intravitreal use [9, 36, 37]. Conversely, infliximab and etanercept have been shown to induce uveitis and retinal toxicity. Intravitreal studies of adalimumab and infliximab by the Pan-American Collaborative Retina Study Group also revealed unfavorable results [7, 9]. 

As a result, in support of the literature, anti-TNF medication seems to reduce the incidence of diabetic retinopathy independent of its glucose-lowering effect. Although the outcomes of direct intravitreal administration studies have been controversial, with the increasing and widespread use of anti-TNFs, their anti-inflammatory role in the pathophysiology of diabetes and its complications has become a hot research topic. This effect can be attributed to its anti-inflammatory role in diabetes pathogenesis. Further prospective studies with larger cohorts are necessary to establish the long-term impact of biologics on the development and treatment of DR.

## Limitations

The primary drawback of our study is its retrospective design; however, the design of a large prospective study to evaluate the intersection of two different complicated disease groups in which many environmental and genetic factors are involved and at the same time, many different biomarkers need to be monitored will only be possible with a nationwide collaboration. Therefore, we believe the relatively limited amount of data available for such a group, which is challenging to match, is valuable.

Another limitation of our study was that we ignored the effect of treatment on patients’ comorbidities. For example, fenofibrate, which is used to treat hypertriglyceridemia, has been reported to reduce retinal inflammation, and randomized controlled trials have shown that this moderate-certainty effect is only at the level of reducing progression in patients with existing retinopathy [38] (Table 3). 

**Table 3:** Univariate and Multivariate regression analysis of risk factors for retinopathy

	Univariate				Multivariate			
	*p*	OR			*p*	OR		
Age	0.090	1.056			0.144	1.077		
Gender (Male)	0.599	1.333			0.589	1.503		
Diabetes duration	0.342	1.034			0.449	1.041		
HBA1C	< 0.001*	2.104			< 0.001*	2.729		
Creatinine	0.020*	22.004			0.582	3.026		
Coronay artery disease	0.278	1.932			0.511	0.543		
Hypertension	0.732	0.789			0.795	0.796		
Hyperlipidemia	0.234	1.944			0.972	0.973		
TNFi (Group-1)	0.012*	0.182			0,013*	0.107		

*: A *p*-value lower than 0.05 was considered significant
